# Recurrent Malignant Melanoma on the Tongue: A Case Report and Review of the Literature

**DOI:** 10.1002/cnr2.70215

**Published:** 2025-05-08

**Authors:** Maziar Motiee‐Langroudi, Athena Farahzadi, Pouyan Aminshakib

**Affiliations:** ^1^ Imam Khomeini Complex Hospital Cancer Institute, Tehran University of Medical Sciences Tehran Iran; ^2^ Division of Surgical Oncology Cancer Institute, Tehran University of Medical Sciences Tehran Iran; ^3^ Pathology Department Cancer Institute, Imam Khomeini Complex Hospital, Tehran University of Medical Sciences Tehran Iran

**Keywords:** mucosal melanoma, recurrent tongue melanoma, tongue malignant melanoma

## Abstract

**Background:**

Melanoma of the oral mucosa is an uncommon cancer arising from the tissues lining the mouth. Among oronasal malignant melanomas, tongue melanoma makes up a mere 2%. Optimal treatments for this rare and often late‐stage disease remain elusive. However, surgery with free margins is considered the primary treatment and is often combined with other therapies such as neck dissection, adjuvant radiotherapy, chemotherapy, and immunotherapy.

**Case:**

This case involves a 33‐year‐old woman with a history of malignant melanoma on her tongue. She had previously undergone a partial glossectomy and was on maintenance imatinib treatment for ~2 years. During her follow‐up, a new lesion was discovered on her tongue, which was confirmed to be malignant melanoma and was resected. The tumor exhibited a depth of invasion of 8 mm. All surgical margins were clear, with the closest margin being 3 mm. The lesion was reconstructed with a submental flap. Adjuvant radiotherapy was also given. The patient has been on maintenance follow‐up for 3 years with no signs of recurrence.

**Conclusion:**

Malignant melanoma should be considered in the differential diagnosis of pigmented and non‐pigmented lesions of the tongue and oral mucosa. A thorough clinical evaluation, followed by histopathological and immunohistochemical examination of any suspicious lesions, is essential for early diagnosis. Early detection and prompt treatment are crucial for optimizing patient outcomes and improving survival rates.

## 1 Introduction

Primary oral mucosal melanoma (OMM) is a very uncommon entity that makes up about 1%–8% of all malignant melanomas and < 0.5% of all oral malignant tumors [1, 2]. It originates from melanocytes in the oral cavity, which are part of the immune system in the mouth [1, 3].

Primary oral mucosal melanoma (OMM) most frequently involves the maxillary gingiva or hard palate, with tongue involvement occurring in under 2% of oronasal melanoma cases [4]. The etiology of OMM is unknown. According to the literature, environmental factors and habits like alcohol consumption, cigarette smoking, denture irritation, and trauma may affect its occurrence [5, 6]. Additionally, individuals with weakened immune systems, including those with conditions like HIV, non‐Hodgkin lymphoma, chronic lymphocytic leukemia, or organ transplants, are more susceptible to developing OMM [5]. While research is ongoing, a family history of melanoma, particularly familial melanoma, may increase the risk of developing OMM.

The majority of OMMs are believed to be De nova [7]. Mucosal melanosis was found to be related to oral malignant melanoma in 66% of cases. It was present before diagnosis in 36.2% of cases and was synchronous in 29.8% of patients, according to the report by Takagi et al. [4]. However, the mechanism behind this transformation is not yet clearly understood [8, 9]. Melanoma development can be influenced by mutations in several genes, such as CDKN2A (p16), CDK4 (found on chromosome 12q15), RB1, CDKN2A (p19), and PTEN/MMAC1 that can be targeted for systemic therapy [10]. Our patient has no risk factors for mucosal melanoma, as a majority of OMMs develop spontaneously.

OMM can be presented as an asymptomatic pigmented macule or nodule in the oral mucosal membrane and in some cases without any pigmentation as an amelanotic lesion. OMM can be challenging due to the possibility of misdiagnosis with other pigmented lesions like melanosis, melanotic macules, oral nevi, racial pigmentation, post‐inflammatory pigmentation, amalgam tattoos, medication melanosis, melanoacanthoma, Peutz–Jeghers syndrome, Addison disease, and Kaposi sarcoma [9]. The primary tumor may be surrounded by satellite foci, and the surface can appear smooth, unbroken, or eroded [2, 10]. Most people do not inspect their oral cavity precisely, even pigmented variants are usually diagnosed late at an advanced stage when they become painful, hemorrhagic, and ulcerative. The clinical manifestations of OMM may differ broadly. According to Tanaka et al.'s classification system, OMM can be categorized into five subtypes: pigmented nodular, pigmented macular, pigmented mixed, non‐pigmented nodular, and non‐pigmented mixed [10]. OMM is presented with various signs and symptoms, including bleeding, poorly fitting dentures, pain, loosening of teeth, and delayed healing of tooth sockets [2].

OMM has a dismal prognosis. It is somewhat related to late diagnosis and advanced stage of the disease. To ensure an accurate diagnosis, a biopsy is advised for any suspicious lesion in the mouth. For small lesions, complete removal with a 1–2 mm margin is necessary. In the case of larger lesions, a sample biopsy should be obtained from the thickest or most concerning area. Hematoxylin and eosin staining can diagnose OMM. Immunohistochemical staining for markers like HMB45 and Melan‐A can be employed to solidify the diagnosis of melanoma [11, 12, 13].

Building on the work of Greene et al. (1953), to diagnose primary oral malignant melanoma, cancer must be confirmed by both microscopic and clinical examination, originate from the junction of the epithelium and connective tissue, and have no other primary cancer site [12].

Early detection and treatment would ameliorate the prognosis remarkably. The best treatment option is not well depicted due to its rarity and poor prognosis. Radical surgery is the “gold standard” treatment of OMM. Depending on the severity of the cancer and lymph node involvement in the neck, neck dissection surgery may be necessary. Non‐surgical treatments like radiotherapy and medications, including checkpoint inhibitors, as additional therapy based on the cancer stage, can also be used as adjuvant treatment [1]. Several factors significantly influence prognosis, including the size and depth of the tumor, the presence of lymphovascular invasion (LVI), necrosis, variations in cancer cells, lymph node involvement, and metastasis [11]. The 5‐year survival rate of OMM is about 15%–38%, which is the lowest among other melanoma [3].

This case report details a rare occurrence of oral melanoma recurrence in a young woman. Despite previous successful treatment for tongue melanoma involving surgery and targeted therapy, the cancer returned in the same location. This highlights the potential for melanoma to recur, even after initial treatment. The case report emphasizes the importance of early diagnosis, especially in younger individuals, as melanoma can present in various forms, including pigmented and non‐pigmented lesions. The successful management of the recurrent melanoma through a combination of surgery, reconstruction, and adjuvant radiotherapy demonstrates the potential effectiveness of this multi‐modal approach.

By documenting this unique case and its successful outcome, the report contributes to the limited body of knowledge on oral melanoma. It provides valuable insights for clinicians in diagnosing and treating this aggressive malignancy, particularly in cases of recurrence.

## 2 Case Report

A 33‐year‐old woman from Tehran presented to our clinic (Cancer Institute, Imam Khomeini Hospital Complex) in September 2019 with a five‐month history of a gradually enlarging, pigmented, and ulcerated mass on her left posterolateral tongue. Upon physical examination, there was a 1 cm pigmented ulcerated mass with a satellite smaller discoloration, without palpable cervical lymphadenopathy. An incisional biopsy revealed that the patient had malignant melanoma. MRI showed an enhanced lesion measuring about 14 mm × 6 mm at the posterolateral part of the tongue without cervical lymphadenopathy. A chest abdominopelvic CT scan revealed no evidence of distant metastasis. The patient had no history of smoking, alcohol use, immunosuppression, or family melanoma. A partial glossectomy was performed with safe margins, and the defect was repaired with a tongue local flap. Pathology revealed malignant melanoma with satellite lesions with a maximum thickness of 5–6 mm, with striated muscle infiltration. There was evidence of macroscopic and microscopic satellite nodules and lymphovascular invasion. All surgical margins were clear. She refused postoperative radiotherapy but was further treated with imatinib (400 mg/daily) due to the strong positivity of C‐KIT without complication.

**FIGURE 1:**
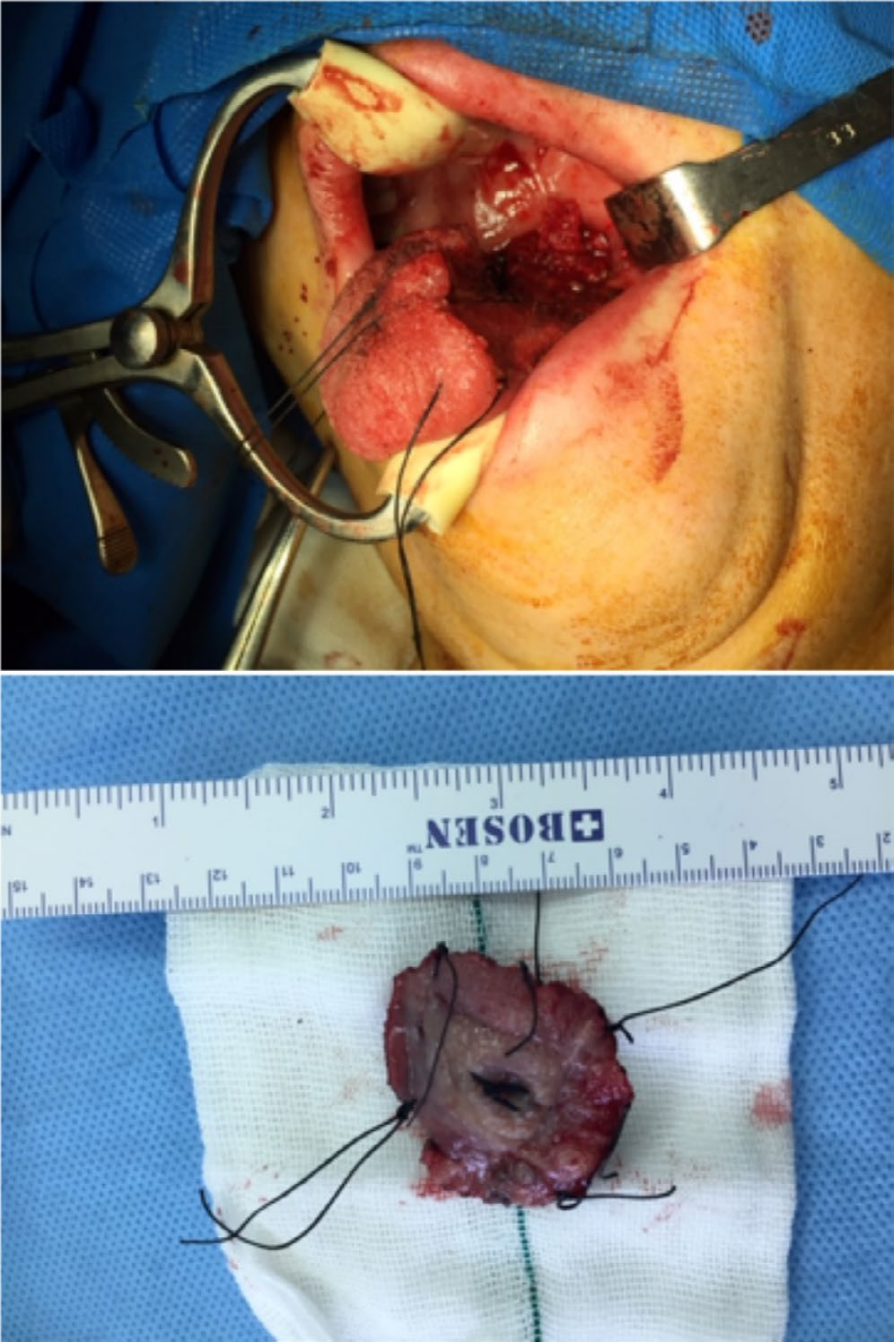
Intraoperative view of the surgical defect in the posterolateral tongue (Top). Resected tongue tissue specimen (Bottom).

**FIGURE 2:**
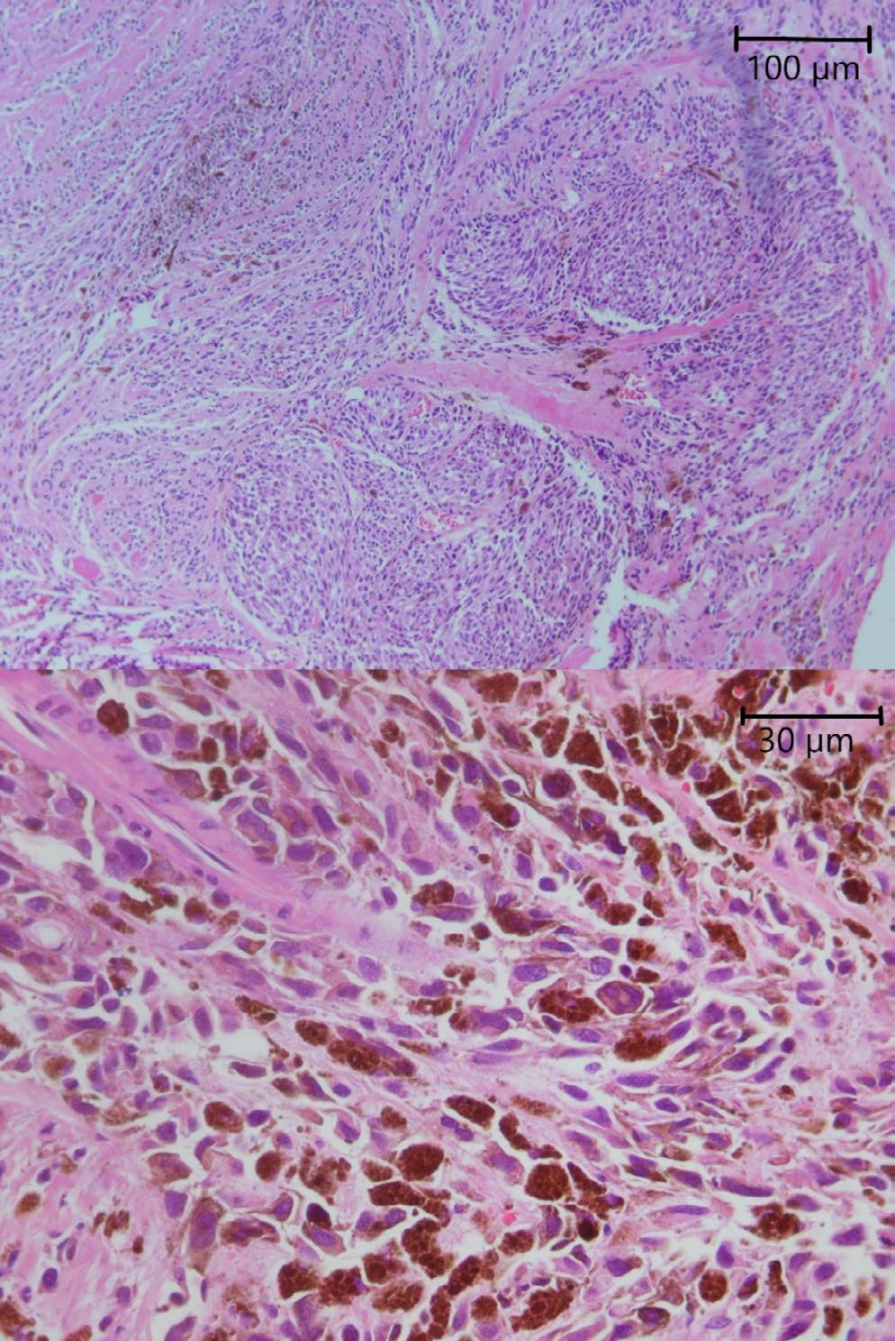
Photomicrographs show infiltrative nests of haphazardly arranged neoplastic cells (A: ×40), demonstrating significant nuclear pleomorphism and prominent aggregation of melanin pigment (B: ×100).

During her regular follow‐up, which consists of clinical visits and physical exams every 3 months, no evidence of recurrence was found for 2 years (October 2021). She noticed a black spot on her tongue that gradually increased in size without accompanying symptoms. During a physical examination, a pigmented mass of size 15 mm × 10 mm was found in the left posterolateral area of the patient's tongue. The lesion was indurated and non‐tender, without ulceration. No other similar lesions were found either in the mouth or other parts of the body. MRI of the neck revealed an enhancing lesion measuring 14.6 mm in the left posterolateral part of the tongue related to the patient's known pathology. An incisional biopsy of the lesion revealed malignant mucosal melanoma. PET scans and neck ultrasonography revealed no evidence of distant metastasis or local adenopathy. Due to the absence of neurological symptoms and a normal PET scan, a brain MRI was not performed. Her condition was discussed on the multidisciplinary tumor board. Due to economic sanctions, limited access to novel targeted therapies and immunotherapies, inadequate insurance coverage for available systemic treatments, and the patient's financial constraints, the tumor board recommended surgery and adjuvant radiotherapy. She underwent a partial glossectomy (Figure 1). The frozen section confirmed that tumor margins were free of malignant cells. A submental flap was used to repair the defect. Prophylactic neck dissection was not done due to a negative neck exam and imaging. Her post‐op course was uneventful. The pathology report revealed T3N0 malignant melanoma with safe margins (Figure 2). The maximum tumor thickness was 8 mm. Ulceration was present. A microsatellite lesion was not identified. There was no evidence of lymphovascular invasion or neurotropism. Mitotic rate was 4 mitoses per mm [2]. Adjuvant radiotherapy was started about a month after surgery (60 Gy in 30 fractions, December 2021). She is currently undergoing regular follow‐ups. No local recurrence of the lesion was found during the 3‐year follow‐up period (until October 2024).

## 3 Discussion

OMM is an uncommon cancer of the mouth lining, representing only 0.2%–0.8% of all malignant melanomas. Weber first documented this rare condition in 1859 [1, 4, 14]. The palate and maxillary gingiva are the predominant sites for intraoral melanoma, accounting for most cases (80%–90%). However, it can also affect any other mucosal site in the oral cavity [1, 2]. Tongue melanoma is extremely rare [5, 14]. OMM typically affects males between ages 40 and 70 at a higher ratio than females [13, 15]. However, there is no gender difference in some studies [4, 15, 16]. It is diagnosed about 10 years earlier in males than in females. It affects races differently, and the Japanese are more susceptible to OMM, which can point to a genetic or environmental tendency that has not been identified yet [4, 17, 18]. We presented a young woman in her 4th decade of life with a history of primary tongue melanoma who presented with recurrent tongue melanoma while on maintenance Imatinib treatment.

The etiology of OMM remains poorly understood, distinct from cutaneous melanoma's (CM's) link to sun exposure. Suspected risk factors, though lacking strong evidence, include tobacco and alcohol use, ill‐fitting dentures, formaldehyde exposure, and potentially genetic predispositions. Specific genetic mutations driving OMM are not as well defined as in CM [19, 20].

OMM carries a poor prognosis, often due to late diagnosis and frequent lymph node and distant metastasis. Prognostic factors include clinical features like tumor size (especially > 1 cm for nodular lesions), ulceration, and neck lymphadenopathy, as well as histological features like Ki67 expression, BAP1 loss, heparanase expression, and potentially depth of invasion. Treatment primarily involves wide surgical excision with negative margins. Adjuvant radiotherapy may be used for local control, even though OMM is generally considered radio resistant. Immunotherapy, particularly immune checkpoint inhibitors, holds promise, though its effectiveness compared with CM requires further investigation. The roles of chemotherapy and targeted therapies are also being explored. Given the aggressive nature of OMM, a multidisciplinary approach is crucial, and ongoing research focuses on improving early detection, understanding the molecular drivers of the disease, and optimizing therapeutic strategies, including immunotherapy and targeted agents [19, 21].

There are several staging systems for malignant mucosal melanoma based on the TNM staging system, micro invasion, histopathologic pattern, and so on. Neither of them is commonly utilized. In 1970, Ballantyne's work established a three‐tier staging system for categorizing mucosal melanomas [22]. This system remains frequently used today, with Stage I being defined as the presence of the primary tumor (T any N0M0) without lymph node involvement or distant metastasis, which is further subdivided into three levels: Level I is non‐invasive melanoma, Level II invades the lamina propria, and Level III penetrates deeper into the underlying tissues. Stage II is regional lymph node involvement (T any N1M0). Stage III indicates distant metastasis (T any N any M1) [2, 13, 22, 23, 24].

The American Joint Committee on Cancer's (AJCC) 9th edition classification for OMM considers stage III the earliest form of the disease (T3, N0). Stage IV is divided into three parts: Stage IV A consists of (T3N1) or (T4a, N0 or N1), Stage IV B (T4b, N0 or N1), and Stage IV C (any T, any N with metastasis) [9, 20, 25] (Table 1).

**TABLE 1:** The American Joint Committee on Cancer (AJCC) staging system (T, Tumor [9th edition]).

T category	T criteria
T3	Confined to the mucosa and the shallow soft tissue directly beneath it, with no limitation on thickness or size
T4	Moderately or highly advanced
T4_a_	Extends beyond the mucosa and into deeper structures, such as deep soft tissue, cartilage, bone, or even the overlying skin
T4_b_	Extends to critical structures such as the brain, dura, skull base, or major blood vessels, nerves (glossopharyngeal, vagus, spinal accessory, and hypoglossal), the masticator space, the prevertebral space, or even structures in the chest cavity

In the case of a suspected tumor, CT and MRI can be used for the evaluation of the loco‐regional extent of the lesion, which is fundamental for defining the resectability of the tumor. MRI scans are generally preferred for a more comprehensive evaluation [22]. German guidelines consider a thorough clinical examination sufficient for staging melanoma in situ. Head MRI, whole‐body imaging like PET‐CT, CT, or skeletal scintigraphy combined with LDH and tumor marker S100B are used in advanced stages [1].

The optimal therapeutic management of OMM remains controversial. Combining various treatment methods might offer a greater advantage for treating mucosal melanoma [19]. Radical surgery with negative margins of the primary lesion is the cornerstone of treatment [15, 22, 26, 27]. The safety margin of the OMM is at least 1.5 cm based on the National Comprehensive Cancer Network (NCCN) like oral cavity SSC, and 2.5 cm for lesions larger than 3 cm [2, 13]. Although complete tumor excision is essential, the extent of safety margins has not been clarified [28]. If wide free margins (like 3 cm) are leading to serious morbidity, the surgeon can decrease them to just a negative margin (like 5 mm) because there is no survival difference considering the size of the safety margin [23, 25, 28].

Neck dissection is generally reserved for situations where preoperative tests confirm lymph node metastasis. Prophylactic neck dissection, performed without evidence of positive lymph nodes, is not recommended due to the minimal impact on survival rates [12, 22, 28, 29, 30, 31].

Sentinel lymph node biopsy (SLNB), which is currently used in CM, is not usually performed at OMM. However, it is technically possible [32, 33, 34]. SLNB can be used to assess the lymph nodes in the neck of individuals with OMM for signs of cancer spread. This may provide another option for identifying patients who would be candidates for elective neck dissection [31, 35, 36, 37]. Research by Starek et al. suggests a connection between finding microscopic cancer cells in a sentinel lymph node and the early spread of cancer through the bloodstream [19, 38]. SLNB is not the standard of care and can be used as a prognostic factor and a potentially efficient staging tool in mucosal melanoma. Although few studies have applied SLNB in OMM, further investigation is warranted [12, 31, 39, 40, 41].

Radiotherapy, chemotherapy, and immunotherapy can be combined with surgery, although their effectiveness is unknown [2, 19]. Poor prognostic pathologic features like multiple positive nodes or extranodal extension are indicators for post‐operative radiotherapy [2, 22, 27]; However, OMM is considered radio‐resistant; it can help to achieve local control despite not improving overall survival [12, 42, 43, 44]. Several factors, including a tumor exceeding 5 mm in thickness, invasion of blood vessels, cell death, variations in tumor cell shape, incomplete removal, location of the primary tumor, invasion of bone, and metastasis, have been linked to worse outcomes for patients with primary oral malignant melanoma (OMM) [2]. Researchers have also pinpointed several independent indicators that can predict a tumor's recurrence. These include the location of the primary tumor in the head or neck, the presence of an ulcer, tumor thickness, a mitotic rate exceeding 3 per square millimeter, positive SLNB, and signs of aggressive tumor growth [32].

OMM exhibits a distinct genetic profile compared with melanomas arising at other sites. While the proto‐oncogene KIT plays a significant role in OMM oncogenesis, as evidenced by the correlation between c‐kit protein expression and activating KIT mutations, these mutations are found in a subset of OMM patients. For instance, Ma et al. detected KIT mutations in 15.8% (22/139) of their OMM cohort and associated these mutations with a poorer prognosis in metastatic disease. Other commonly mutated genes in CM, such as BRAF and NRAS, are less frequently affected in OMM. Thuaire et al.'s review reported BRAF mutations in 7.0% and NRAS mutations in 5.6% of OMM cases, significantly lower than the ~50% and ~30% observed in CM, respectively. Similarly, TERT promoter mutations, common in CM (~48%), appear to be rare or absent in OMM. Miao et al. found no TERT promoter mutations in their study of 39 OMM cases, although Lyu et al. reported a recurrently amplified region containing the TERT promoter. Finally, GNAQ and GNA11 mutations, which occur in only 2%–4% of CM, are also infrequent or absent in OMM. These genetic differences highlight the distinct molecular landscape of OMM compared with CM and have implications for targeted therapies [45]. After identifying specific genetic mutations in the tumor, it is possible to tailor systemic therapy using drugs that target these mutations, such as BRAF, MEK, KIT inhibitors, or checkpoint inhibitors. This can help extend overall survival while keeping the rate of side effects at an acceptable level [1, 22]. The c‐KIT gene plays a vital role in controlling the growth, development, movement, and reproduction of melanocytes. Research has shown that around 80% of mucosal melanoma cases harbor activating mutations in this gene, suggesting its potential involvement in this specific type of cancer. This pathway is momentous in melanoma and is not related to sun exposure [25]. The RAS/MEK/ERK pathway, also known as the mitogen‐activated protein kinase (MAPK) pathway, is critical for the growth of OMM. Immunotherapy offers a promising treatment option for aggressive forms of malignant melanoma, including those with a high risk of recurrence and metastatic spread, with minimal side effects [12, 20, 35].

Surgical procedures can salvage up to 25% of patients with local recurrence. In patients with recurrent diseases and without distant metastasis like our patient, a second surgery appears to be the most favorable course of action if the lesion can be completely resected without considerable morbidity. Studies have shown that surgical intervention can potentially improve outcomes for up to a quarter (25%) of patients experiencing a local recurrence [35].

Oral melanomas are more aggressive and have a higher tendency for metastasis than other oral cancers or CMs [8, 12]. Several factors contribute to the aggressive nature of oral malignant melanoma (OMM). These include blood vessel invasion, tumor location that makes complete surgical removal difficult, delayed diagnosis, and a propensity for early ulceration due to frequent irritation. Ulceration can create pathways for cancer cells to spread to nearby lymph nodes and distant parts of the body, leading to a higher risk of metastasis [13, 28]. About 25% of patients with OMM present with lymph node metastasis [2, 12, 35].

Delayed diagnosis is often associated with advanced tumors that have already metastasized [14]. After surgical removal of the primary tumor, recurrence and metastasis are common. As a result, most patients succumb to the disease within 2 years. Studies report a wide range for the 5‐year survival rate, from 4.5% to 48%, with most falling between 10% and 25% [4].

## 4 Conclusion

This case report highlights the rarity and challenges associated with oral melanoma, specifically on the tongue. The primary takeaways are that due to the potential for late diagnosis, clinicians should maintain a high index of suspicion for oral melanoma, even in the absence of pigmentation. Thorough clinical examination, along with histopathological and immunohistochemical analysis, is vital for accurate diagnosis. Surgical resection with clear margins is the primary treatment, but adjuvant therapies like radiotherapy and immunotherapy may be beneficial. Given the risk of recurrence, close and prolonged follow‐up is essential for patients with oral melanoma.

### 4.1 Timeline

A timeline of a 33‐year‐old woman with malignant melanoma of the tongue.
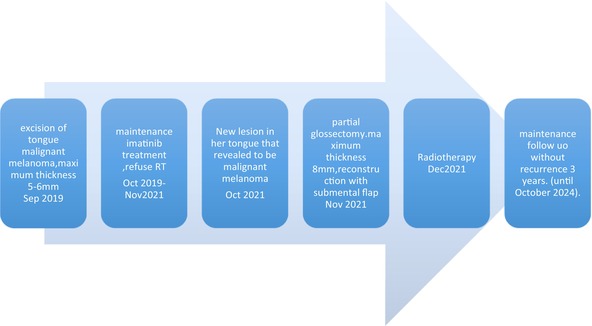


## Author Contributions

**Athena Farahzadi:** conceptualization, design, data collection and processing, literature review, writing, and editing. **Maziar Motiee‐Langroudi:** conceptualization, writing, and editing. **Pouyan Aminshakib:** writing and editing.

## Ethics Statement

This report adheres to the journal's patient consent policy, with the patient's written authorization for publication secured.

## Consent

Written informed consent was obtained from the patient to publish this report in accordance with the journal's patient consent policy.

## Conflicts of Interest

The authors declare no conflicts of interest.

## Supporting information


**Data S1.** Supporting Information.

**Data S2.** Supporting Information.

## Data Availability

The data that support the findings of this study are available on request from the corresponding author. The data are not publicly available due to privacy or ethical restrictions.

## References

[1] P. Becker , A. Pabst , M. Bjelopavlovic , D. Müller , and P. W. Kämmerer , “Treatment Modalities of Recurrent Oral Mucosal Melanoma In Situ,” Medicina 57 (2021): 965, 10.3390/medicina57090965. 34577888 PMC8469538

[2] N. Sharma , “Primary Oral Malignant Melanoma: Two Case Reports and Review of Literature,” Case Reports in Dentistry 2012 (2012): 975358, 10.1155/2012/975358. 22900212 PMC3414006

[3] H. Tyrrell and M. Payne , “Combatting Mucosal Melanoma: Recent Advances and Future Perspectives,” Melanoma Management 5, no. 3 (2018): MMT11, 10.2217/mmt-2018-0003. 30459941 PMC6240847

[4] H. Khalifa , S. Abdullah , K. Sallam , H. Khalil , I. Abdel Moneim , and A. Elaffandi , “Primary Malignant Melanoma of the Tongue,” Canadian Journal of Surgery 52, no. 6 (2009): E309–11. PMC2792379 20011176

[5] D. Singh , P. Pandey , M. K. Singh , and S. Kudva , “Prevalence of Malignant Melanoma in Anatomical Sites of the Oral Cavity: A Meta‐Analysis,” Journal of Oral and Maxillofacial Pathology 23 (2019): 129–135. 31110429 10.4103/jomfp.JOMFP_236_18 PMC6503777

[6] J. J. Lee , L. Y. Wei , Y. C. Wu , and C. P. Chiang , “Oral Tongue Melanoma,” Journal of the Formosan Medical Association 112, no. 11 (2013): 730–731, 10.1016/j.jfma.2013.09.011. 24144529

[7] M. Venugopal , I. Renuka , G. S. Bala , and N. Seshaiah , “Amelanotic Melanoma of the Tongue,” Journal of Oral and Maxillofacial Pathology 17, no. 1 (2013): 113–115, 10.4103/0973-029X.110699. 23798843 PMC3687165

[8] M. M. Mahmoud , M. Madi , and R. Gamal , “Uncommon Clinical Presentation of Oral Malignant Melanoma,” Beni‐Suef University Journal of Basic and Applied Sciences 7, no. 2 (2018): 220–222, 10.1016/j.bjbas.2017.11.003.

[9] R. Aloua , A. Kaouani , O. Kerdoud , I. Salissou , and F. Slimani , “Melanoma of the Oral Cavity: A Silent Killer,” Annals of Medicine and Surgery (London) 62 (2021): 182–185, 10.1016/j.amsu.2021.01.026. PMC7829076 33532067

[10] B. Collins , J. Abernethy , and L. Barnes , Oral Malignant Melanoma (Oral Cancer Foundation, 2010), https://oralcancerfoundation.org/facts/rare/oral‐malignant‐melanoma.

[11] I. J. Dias , I. J. Ferreira Filho , J. V. Pereira , et al., “Melanoma of the Oral Mucosa: Report of an Aggressive Case and Review of the Literature,” Journal of Oral Diagnosis 2 (2017): 1–6, 10.5935/2525-5711.20170041.

[12] N. Sharma Lamichhane , J. An , Q. Liu , and W. Zhang , “Primary Malignant Mucosal Melanoma of the Upper Lip: A Case Report and Review of the Literature,” BMC Research Notes 8 (2015): 499, 10.1186/s13104-015-1459-3. 26420268 PMC4589098

[13] P. Gadodia , R. Wadhwani , A. Dhobley , N. Patil , V. K. Patil , and V. Murgod , “Malignant Melanoma: A Case Report With Literature Review,” New Jersey Institute of Technology Research and Innovation Magazine 7, no. 1 (2016): 120–124.

[14] G. M. Gu , J. B. Epstein , and T. H. Morton, Jr. , “Intraoral Melanoma: Long‐Term Follow‐Up and Implication for Dental Clinicians: A Case Report and Literature Review,” Oral Surgery, Oral Medicine, Oral Pathology, Oral Radiology, and Endodontics 96 (2003): 404–413. 14561964 10.1016/s1079-2104(03)00320-2

[15] N. Mellouli , S. Sioud , M. Garma , A. Chokri , H. Hamdi , and J. Selmi , “Oral Malignant Melanoma: History of Malignant Degeneration of a Pigmented Lesion,” Journal of Oral Medicine and Oral Surgery 25 (2019): 19.

[16] F. Ahmadi‐Motamayel , P. Falsafi , and F. Baghaei , “Report of a Rare and Aggressive Case of Oral Malignant Melanoma,” Oral and Maxillofacial Surgery 17 (2013): 47–51. 22367683 10.1007/s10006-012-0311-3 PMC3576568

[17] M. Takagi , G. Ishikawa , and W. Mori , “Primary Malignant Melanoma of the Oral Cavity in Japan. With Special Reference to Mucosal Melanosis,” Cancer 34 (1974): 358–370. 4853771 10.1002/1097-0142(197408)34:2<358::aid-cncr2820340221>3.0.co;2-d

[18] S. P. Bansal , S. S. Dhanawade , A. S. Arvandekar , V. Mehta , and R. S. Desai , “Oral Amelanotic Melanoma: A Systematic Review of Case Reports and Case Series,” Head and Neck Pathology 16, no. 2 (2021): 513–524, 10.1007/s12105-021-01366-w. 34309791 PMC9187796

[19] B. Barata , F. Freitas , M. Vilares , and J. Caramês , “Oral Mucosal Melanoma: A Systematic Review of Case Reports and Case Series,” Journal of Oral and Maxillofacial Surgery, Medicine, and Pathology 36, no. 3 (2024): 388–395, 10.1016/j.ajoms.2023.09.002.

[20] American Joint Committee on Cancer , Cancer Staging Manual, 9th ed. (Springer, 2023).

[21] Y. J. Jeong , J. F. Thompson , and S. Ch'ng , “Epidemiology, Staging and Management of Mucosal Melanoma of the Head and Neck: A Narrative Review,” Chinese Clinical Oncology 12, no. 3 (2023): 28, 10.21037/cco-23-16. 37417292

[22] U. de Bezerra Toscano Mendonça , J. Guimarães Soffientini , V. Ficher Barbosa , and K. Cozer , Mucosal Melanoma of the Head and Neck: From Diagnosis to Treatment (IntechOpen, 2021), 10.5772/intechopen.93804.

[23] P. Pradhan and A. K. Adhya , “Extensive Malignant Melanoma of the Oral Cavity: A Rare Occurrence,” Autopsy & Case Reports 11 (2021): 2021299, 10.4322/acr.2021.299. PMC8387067 34458169

[24] P. M. Zito and T. Mazzoni , Oral Melanoma (StatPearls Publishing, 2021). 30020648

[25] Y. Lee and E. I. Auerkari , “A Study of Eight Oral Malignant Melanoma in Adults by WESTOP (Western Society of Teachers of Oral Pathology, 1995),” Journal of Hard Tissue Biology 14 (2005): 122, 10.2485/jhtb.14.122.

[26] T. T. Chiu , H. C. Lin , C. Y. Su , and C. C. Huang , “Primary Malignant Melanoma of the Tongue,” Chang Gung Medical Journal 25, no. 11 (2002): 764–768. 12553365

[27] L. Pincet , K. Lambercy , P. Pasche , M. Broome , S. Latifyan , and A. Reinhard , “Mucosal Melanoma of the Head and Neck: A Retrospective Review and Current Opinion,” Frontiers in Surgery 7 (2021): 616174, 10.3389/fsurg.2020.616174. 33585548 PMC7873938

[28] Y. S. Chae , J. Y. Lee , J. W. Lee , J. Y. Park , S. M. Kim , and J. H. Lee , “Survival of Oral Mucosal Melanoma According to Treatment, Tumour Resection Margin, and Metastases,” British Journal of Oral & Maxillofacial Surgery 58, no. 9 (2020): 1097–1102, 10.1016/j.bjoms.2020.05.028. 32586691

[29] R. J. Patrick , N. A. Fenske , and J. L. Messina , “Primary Mucosal Melanoma,” Journal of the American Academy of Dermatology 56, no. 5 (2007): 828–834, 10.1016/j.jaad.2006.06.017. 17349716

[30] M. Pittaka , D. Kardamakis , and D. Spyropoulou , “Comparison of International Guidelines on Mucosal Melanoma of the Head and Neck: A Comprehensive Review of the Role of Radiation Therapy,” In Vivo 30, no. 3 (2016): 165–170. 27107071

[31] M. S. Oldenburg and D. L. Price , “The Utility of Sentinel Node Biopsy for Sinonasal Melanoma,” Journal of Neurosurgery 78, no. 5 (2017): 425–429, 10.1055/s-0037-1603960. PMC5582961 28875122

[32] N. B. Seim , C. L. Wright , and A. Agrawal , “Contemporary Use of Sentinel Lymph Node Biopsy in the Head and Neck,” World Journal of Otorhinolaryngology – Head and Neck Surgery 2, no. 2 (2016): 117–125, 10.1016/j.wjorl.2016.05.008. 29204556 PMC5698522

[33] D. H. Kim , Y. Kim , S. W. Kim , and S. H. Hwang , “Usefulness of Sentinel Lymph Node Biopsy for Oral Cancer: A Systematic Review and Meta‐Analysis,” Laryngoscope 131, no. 2 (2021): E459–E465, 10.1002/lary.28728. 32401367

[34] R. R. Clark , T. Shoaib , D. S. Soutar , et al., “Sentinel Lymph Node Biopsy in Oral Malignant Melanoma—A Possible Means of Investigating the Clinically Node‐Negative Neck,” European Journal of Plastic Surgery 28, no. 6 (2005): 403–407, 10.1007/s00238-005-0798-z.

[35] F. López , J. P. Rodrigo , A. Cardesa , et al., “Update on Primary Head and Neck Mucosal Melanoma,” Head & Neck 38, no. 1 (2016): 147–155, 10.1002/hed.23872. 25242350 PMC4986507

[36] G. L. Ross , D. S. Soutar , D. Gordon MacDonald , et al., “Sentinel Node Biopsy in Head and Neck Cancer: Preliminary Results of a Multicenter Trial,” Annals of Surgical Oncology 11, no. 7 (2004): 690–696, 10.1245/ASO.2004.09.001. 15197011

[37] İ. Kaya , S. Göde , K. Öztürk , et al., “The Value of Sentinel Lymph Node Biopsy in Oral Cavity Cancers,” Turkish Archives of Otorhinolaryngology 53, no. 2 (2015): 62–66, 10.5152/tao.2015.1178. 29391982 PMC5783002

[38] I. Stárek , P. Koranda , and P. Benes , “Sentinel Lymph Node Biopsy: A New Perspective in Head and Neck Mucosal Melanoma?,” Melanoma Research 16, no. 5 (2006): 423–427, 10.1097/01.cmr.0000222603.57932.b6. 17013091

[39] A. G. Shuman , E. Light , S. H. Olsen , et al., “Mucosal Melanoma of the Head and Neck: Predictors of Prognosis,” Archives of Otolaryngology – Head & Neck Surgery 137, no. 4 (2011): 331–337, 10.1001/archoto.2011.46. 21502471

[40] P. Baptista , M. J. Garcia Velloso , F. Salvinelli , and M. Casale , “Radioguided Surgical Strategy in Mucosal Melanoma of the Nasal Cavity,” Clinical Nuclear Medicine 33, no. 1 (2008): 14–18, 10.1097/RLU.0b013e31815c5092. 18097249

[41] R. R. Clark and T. Shoaib , “Sentinel Lymph Node Biopsy: A New Perspective in Head and Neck Mucosal Melanoma?,” Melanoma Research 17, no. 1 (2007): 59, 10.1097/CMR.0b013e328014162e. 17235244

[42] A. Trotti and L. J. Peters , “Role of Radiotherapy in the Primary Management of Mucosal Melanoma of the Head and Neck,” Seminars in Surgical Oncology 9, no. 3 (1993): 246–250. 8516612

[43] C. J. I. Caspers , E. A. C. Dronkers , D. Monserez , M. H. Wieringa , R. J. de Baatenburg Jong , and J. A. U. Hardillo , “Adjuvant Radiotherapy in Sinonasal Mucosal Melanoma: A Retrospective Analysis,” Clinical Otolaryngology 43, no. 2 (2018): 617–623, 10.1111/coa.13033. 29150980

[44] O. Azem , O. Nabulsi , M. Jelinek , and N. Joshi , “Radiation Therapy in the Management of Head and Neck Mucosal Melanoma,” Cancers (Basel) 16, no. 19 (2024): 3304, 10.3390/cancers16193304. 39409922 PMC11475939

[45] A. Thuaire , R. Nicot , M. Boileau , et al., “Oral Mucosal Melanoma – A Systematic Review,” Journal of Stomatology Oral and Maxillofacial Surgery 123, no. 5 (2022): e425–e432, 10.1016/j.jormas.2022.02.002. 35134590

