# Supplementary material for: Recurrent Malignant Melanoma on the Tongue: A Case Report and Review of the Literature
Source: Cancer Rep (Hoboken). 2025 May 8;8(5):e70215. doi: 10.1002/cnr2.70215 (PMC12062517; doi:10.1002/cnr2.70215)
Supplement: Supplementary file 1 — Data S1. Supporting Information. [file CNR2-8-e70215-s001.docx]

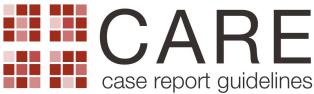
**CARE Checklist (2013) of information to include when writing a case report
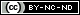
**

**Topic Item Checklist item description Reported on Page**

**Title 1** The words “case report” should be in the title along with the area of focus

**Key Words 2** 2 to 5 key words that identify areas covered in this case report

**Abstract 3a** Introduction—What is unique about this case? What does it add to the medical literature?

**3b** The main symptoms of the patient and the important clinical findings

**3c** The main diagnoses, therapeutics interventions, and outcomes

**3d** Conclusion—What are the main “take-away” lessons from this case?

**Introduction 4** One or two paragraphs summarizing why this case is unique with references

**Patient Information 5a** Demographic information and other patient specific information

**5b** Main concerns and symptoms of the patient

**5c** Medical, family, and psychosocial history including relevant genetic information (also see timeline).

**5d** Relevant past interventions and their outcomes

**Clinical Findings 6** Describe the relevant physical examination (PE) and other significant clinical findings

**Timeline 7** Important information from the patient’s history organized as a timeline

# Diagnostic Assessment

**Therapeutic Intervention**

**Follow-up and Outcomes**

**8a** Diagnostic methods (such as PE, laboratory testing, imaging, surveys)

**8b** Diagnostic challenges (such as access, financial, or cultural)

**8c** Diagnostic reasoning including other diagnoses considered

**8d** Prognostic characteristics (such as staging in oncology) where applicable

**9a** Types of intervention (such as pharmacologic, surgical, preventive, self-care)

**9b** Administration of intervention (such as dosage, strength, duration)

**9c** Changes in intervention (with rationale)

**10a** Clinician and patient-assessed outcomes (when appropriate)

**10b** Important follow-up diagnostic and other test results

**10c** Intervention adherence and tolerability (How was this assessed?)

**10d** Adverse and unanticipated events

**Discussion 11a** Discussion of the strengths and limitations in your approach to this case

**11b** Discussion of the relevant medical literature

**11c** The rationale for conclusions (including assessment of possible causes)

v

**11d** The primary “take-away” lessons of this case report

**Patient Perspective 12** When appropriate the patient should share their perspective on the treatments they received

**Informed Consent 13** Did the patient give informed consent? Please provide if requested . . . . . . . . . . . . . . . . . . . . . . . . . . . . . . . ………… **Yes**
