# Supplementary material for: Recurrent Malignant Melanoma on the Tongue: A Case Report and Review of the Literature
Source: Cancer Rep (Hoboken). 2025 May 8;8(5):e70215. doi: 10.1002/cnr2.70215 (PMC12062517; doi:10.1002/cnr2.70215)
Supplement: Supplementary file 2 — Data S2. Supporting Information. [file CNR2-8-e70215-s002.docx]

Details on the submental flap

The reconstruction of soft tissue defects in the oral cavity is a complex surgical challenge. While microvascular free flaps are considered the gold standard, they have limitations such as high costs, requiring specialized surgical teams, longer surgery and hospital stays.

Locoregional flaps offer a viable alternative, especially when free flaps are not ideal or contraindicated. The submental artery island flap (SAIF) has emerged as a reliable reconstructive option in head and neck surgery. This flap is harvested from the submental region, utilizing the submental artery as its primary blood supply. The submental artery, a branch of the facial artery, courses medially and anteriorly between the submandibular gland and the mylohyoid muscle. It typically travels either deep or superficial to the anterior belly of the digastric muscle, ending behind the mandibular symphysis. Along its course, cutaneous perforators pierce the platysma and anterior belly of the digastric muscles to form a subdermal plexus, allowing for extensive anastomoses with the contralateral artery. The submental vein, which drains into the facial vein, communicates with both the internal and external jugular veins, providing additional venous drainage.

To harvest the SAIF, a curved incision is made in the submental area, extending from below the chin to behind the jawline on both sides. The size of the skin flap is determined by the size of the defect to be repaired and the ability to close the donor site without tension. The flap can be designed to include both sides of the neck if needed, allowing for coverage of larger defects. The upper incision is typically made 1.5 cm below the mandible in the midline and 3.5 cm below the angles of the mandible on both sides. The maximal width of the flap is determined by a pinch ^1^ test to ensure primary closure of the donor site. The length of the flap can vary depending on the specific needs of the reconstruction, with a maximum length spanning from one mandibular angle to the other.

Reference:

Amin, A.A., Sakkary, M.A., Khalil, A.A. *et al.* The submental flap for oral cavity reconstruction: Extended indications and technical refinements. *Head Neck Oncol* **3**, 51 (2011). <https://doi.org/10.1186/1758-3284-3-51>

Amin AA, Sakkary MA, Khalil AA, Rifaat MA, Zayed SB. The submental flap for oral cavity reconstruction: extended indications and technical refinements. Head Neck Oncol. 2011 Dec 20;3:51. doi: 10.1186/1758-3284-3-51. PMID: 22185515; PMCID: PMC3285538.

Ramirez AT, Chiesa-Estomba CM, González-García JÁ. Submental Artery Island Flap in Oral Cavity Reconstruction. An Observational, Retrospective Two-centre Study. Int Arch Otorhinolaryngol. 2021 Jan;25(1):e71-e76. doi: 10.1055/s-0040-1709115. Epub 2020 Apr 24. PMID: 33542754; PMCID: PMC7851373.
